# Expectations of Social Inclusion and Exclusion

**DOI:** 10.3389/fpsyg.2017.00112

**Published:** 2017-02-06

**Authors:** Eric D. Wesselmann, James H. Wirth, Michael J. Bernstein

**Affiliations:** ^1^Department of Psychology, Illinois State UniversityNormal, IL, USA; ^2^Department of Psychology, The Ohio State University at NewarkNewark, OH, USA; ^3^Psychological and Social Science Program, Penn State University-AbingtonAbington, PA, USA

**Keywords:** social exclusion, social inclusion, rejection, ostracism, sociometer theory, relational evaluation

Individuals engage in social interactions generally expecting inclusion (Kerr and Levine, 2008; Wesselmann et al., 2010, 2013). This expectation seems reasonable, given individuals' basic need to establish and maintain social connections to sustain physical and psychological well-being (Baumeister and Leary, 1995). However, individuals often experience *social exclusion*; situations broadly involving someone being disengaged or separated from others physically or emotionally (Riva and Eck, 2016). Exclusion experiences include various phenomena, such as interpersonal rejection, ostracism, and various types of discrimination (Smart Richman and Leary, 2009; Wesselmann et al., 2016).

These diverse threats to social inclusion are so detrimental, researchers argue that humans likely developed mechanisms safeguarding social inclusion (Lieberman, 2013), facilitating quick detection of threats to inclusionary status (Pickett and Gardner, 2005; Kerr and Levine, 2008; Wesselmann et al., 2012b). When threats occur, individuals experience cognitive and behavioral changes to facilitate recovery (Smart Richman and Leary, 2009; Williams, 2009). Considerable research has examined individuals' responses to exclusion, but less has focused on how expectations of inclusion or exclusion moderate those responses. In this article, we highlight research focused on how individuals calibrate their expectations of social inclusion and exclusion, and how these expectations influence the effect of exclusion on individuals' feelings of relational value and other adverse effects of social exclusion.

## Perceiving exclusionary cues

Individuals monitor their environment for exclusionary cues using their *sociometer*, which detects fluctuations in an individual's relational evaluation (Leary, 1999; Leary and Baumeister, 2000). Relational evaluation is operationalized as “the degree to which others regard their relationship with the individual as valuable, important, or close” (Leary, 1999, p. 33). Individuals' perceived relational value is a proxy for inclusionary status (Leary, 1999; Leary and Baumeister, 2000). Exclusionary cues vary from direct to subtle (e.g., language, facial expression, non-verbal behaviors; Kerr and Levine, 2008), yet all produce feelings of social pain (Williams, 2009). Such exclusionary cues may be unambiguously clear, such as a partner stating they do not want to work with you (Maner et al., 2007), not being included during a game (Williams et al., 2000), or being treated in a cold and aloof manner (Geller et al., 1974; Wesselmann et al., 2010). Conversely, exclusion can occur in various subtle ways, such as not receiving eye contact from an avatar, which causes feelings of exclusion (Böckler et al., 2014) and lowered implicit self-esteem (Wirth et al., 2010). Even being stared *through* by a passerby (as if one does not exist) causes feelings of social disconnection (Wesselmann et al., 2012a).

Conversation dynamics can provide cues to one's inclusionary status (Koudenburg, 2014). Smooth conversations indicate relationship solidarity, while uncomfortable pauses are threatening to social connectedness (Koudenburg et al., 2011, 2013). Exclusion can occur during conversations when group members switch to a language unfamiliar to the target (Hitlan et al., 2006; Dotan-Eliaz et al., 2009), when others use unknown acronyms (Hales et al., in press), through exclusive laughter (Klages and Wirth, 2014), or when the conversation makes people feel “out-of-the-loop”—when a person is included in the group, but feels excluded due to knowing there is information that they lack (Jones et al., 2009, 2011).

## When expectations of inclusion are violated

During exclusion, relational devaluation occurs, and individuals suffer aversive physical and psychological consequences (Williams, 2009). However, little work has examined how *expectations* of inclusion/exclusion affect exclusion's consequences. Does expecting exclusion temper the negative outcomes, and unexpected exclusion intensify them, perhaps by threatening individuals' confidence in their sociometers? Because individuals monitor their environments for inclusion-relevant social cues (Leary, 1999; Williams, 2009), they likely experience unexpected exclusion more extremely than excepted exclusion. For example, Wesselmann et al. (2010) found that although excluded individuals aggressed more than included individuals, individuals who experienced *unexpected* exclusion demonstrated the most aggression and showed the least confidence in their sociometer. Further, Wirth et al. (2017) found participants who were unexpectedly excluded experienced increased basic need threat and negative affect, as well as decreased confidence in their sociometer, compared to participants who expected their exclusion. The latter group experienced need threat and negative affect once they received exclusionary social cues, and these negative effects continued on after they were ultimately excluded. Additionally, individuals who expected exclusion did not indicate decreased confidence in their sociometer between the time they received exclusionary cues and when they were excluded. Finally, Rudert and Greifeneder (2016) explicitly manipulated participants' expectations of situational norms and found that excluded participants experienced less negative effects when perceiving exclusion (rather than inclusion) as the norm. Collectively, these studies support neuroscience research suggesting that exclusion-related pain partially involves expectation violations (Somerville et al., 2006).

Based on previous theory (Leary, 1990; Wesselmann et al., 2016), relational evaluation is a key mechanism in understanding the degree to which social exclusion causes negative psychological outcomes. Specifically, deflated relational evaluation can cause negative feelings (Leary et al., 2001; Buckley et al., 2004) and may be related to lowered fulfillment of psychological needs, implicit self-esteem, and aggressive behavior temptations (Wirth et al., 2010; Bernstein et al., 2013). In response, individuals engage in behaviors aimed at safeguarding their relational evaluation. Socially excluded individuals have enhanced memory for social information (Gardner et al., 2000), increased desire to make new friends, and preferences for new potential interaction partners (Maner et al., 2007). Further, excluded individuals show increased attention to genuine signals of social inclusion (Bernstein et al., 2008, 2010a) and emotional expressions of happiness vs. anger (Sacco et al., 2011). Excluded individuals may be guided perceptually and behaviorally toward sources of social inclusion (i.e., increased attunement to positive, inclusive targets; DeWall et al., 2009). To our knowledge, no studies have directly assessed whether relational evaluation mediates excluded participants' perceptual and behavioral biases toward re-inclusion, but some evidence suggests participants' threatened need for belonging can mediate these effects (Bernstein et al., 2010a).

## Future research questions

### Social cue attention and response as adaptation

Research on cognitive responses to exclusion are mixed: some studies show cognitive depletion (Baumeister et al., 2002), whereas others show cognitive benefits such as increased attention to and memory for social information (e.g., Gardner et al., 2000; Pickett et al., 2004; Bernstein et al., 2008). This apparent contradiction may be due to the paradigm used to examine the effects; paradigms revealing deficits tend to involve non-social tasks, while paradigms involving social tasks typically reveal benefits post-exclusion. Perhaps excluded individuals allocate available cognitive resources to tasks most effective to restoring relational evaluation levels, which non-social tasks may not do (Shilling and Brown, 2016).

To our knowledge, research has not directly tested this strategic re-distribution hypothesis (but see Gardner et al., 2000 comparing social and non-social memory). Such studies would strengthen the theoretical argument that individuals respond to exclusion in adaptive ways (i.e., survival-enhancing: Wesselmann et al., 2012b). Additionally, research should investigate if and how expectation violations influence any strategic re-distribution patterns. Williams (2009) argues that once individuals experience the immediate negative effects of exclusion, they subsequently focus cognitive resources on interpreting the situation to assess methods of recovery. These efforts may involve attributional processes or behavioral strategies. If individuals unexpectedly experience decreased relational evaluation, they may show strategic re-distribution more intensely than excluded individuals who expected exclusion because they experienced a more intense threat. Alternatively, simply experiencing *any* exclusion may trigger a strategic re-distribution response that is broad and undifferentiated to maximize re-inclusion efforts (Pickett and Gardner, 2005), and expectations may have little (or no) influence pattern.

Future research should examine if and how expectations matter for chronically excluded individuals. Williams (2009) refers to these individuals as being in the *resignation* stage, and argues that they likely come to expect exclusion in daily interactions. These individuals may experience learned helplessness that effectively comes from being unable to avoid exclusion or alter its consequences. Even though resigned individuals may anticipate exclusion, they may find unexpected exclusionary episodes (either in daily life or in a laboratory setting) more painful than other individuals precisely because they are caught unaware. Alternatively, resigned individuals may simply be numb to the negative effects of exclusion regardless of their momentary expectations (Bernstein and Claypool, 2012; Riva et al., 2014). The resignation stage of exclusion is relatively new and research is sparse (but see Riva et al., 2016), so we can merely speculate on the influence of expectations in this context.

### Paradigm constraints, expectation cues, and responses

Exclusion paradigms often blindside participants with exclusion (Williams and Wesselmann, 2011), but many exclusion experiences outside the laboratory likely involve some warning (Spoor and Williams, 2007; Kerr and Levine, 2008) or clear attributional information relevant during reflection (Nezlek et al., 2012). Thus, the conclusions drawn from most exclusion research may be limited in how well the research represents these everyday exclusion experiences. We have already discussed how Wirth et al. (2017) showed that the social cues prior to exclusion can affect individuals' expectations of, and ultimately responses to, their exclusion. Tuscherer et al. (2016) examined an additional understudied factor, participants' perceptions of fairness for an exclusion experience, and found that participants who perceived their exclusion as unfair experienced greater threat to efficacy needs (i.e., control and meaningful existence) than participants who perceived their exclusion as fair. These studies suggest that researchers should be mindful of how these two factors may relate to their future research questions and design their paradigms accordingly. Researchers should also consider how social cues and perceptions of fairness may influence each other both within a single exclusion episode and across subsequent episodes.

Participants' expectations of inclusion also likely influence *whether* participants will choose to respond pro- or anti-socially to exclusion, as well as the *degree* of their response, which is a current paradox in the literature (Wesselmann et al., 2015). Some research demonstrates that excluded participants will only respond pro-socially when they perceive the opportunity for re-affiliation (Maner et al., 2007; Mead et al., 2011). Potentially, any exclusion paradigm could be adapted to influence participants' expectations by manipulating explicit situational norms (Rudert and Greifeneder, 2016), confederate social cues in face-to-face or virtual *get-acquainted* paradigms (Wesselmann et al., 2010; Wirth et al., 2017), or explicit instructions involving opportunities to meet the target of participants' pro-/anti-social behavior (Maner et al., 2007). Researcher could also use the *life alone* paradigm (Twenge et al., 2001), which provides participants with fake feedback about their future social lives (e.g., their future will be lonely), as an expectations manipulation and then examine how those expectations influence the effects of subsequent exclusion using *in vivo* paradigms.

### Further integrating relational evaluation into research

Researchers should investigate the specific role that relational evaluation plays in the consequences of exclusion, and how situation-level and individual-level characteristics influence this construct. *Situational factors* that may influence expectations of relational evaluation could be the psychological closeness of the sources of inclusion (e.g., a romantic partner; Arriaga et al., 2014), exclusion by in-group vs. out-group members (e.g., Gonsalkorale and Williams, 2007; Bernstein et al., 2010b; Goodwin et al., 2010; Cursan et al., 2016), or situations that require some type of exclusion (i.e., *role-based* exclusion, Nezlek et al., 2012; Rudert and Greifeneder, 2016); although exclusion in each case may hurt, violations of one's expected relational evaluation may help explain if and when exclusion may hurt more (or less) initially, and may also explain differential recovery (e.g., Wirth and Williams, 2009). *Individual factors* may also influence one's expected relational evaluation levels. For example, narcissistic individuals may expect high relational evaluation and thus respond with more aggression than non-narcissists when their expectations are violated (Bushman and Baumeister, 1998; Twenge and Campbell, 2003). Additionally, individuals high in rejection sensitivity (Downey and Feldman, 1996) may have lower expectations for perceived relational evaluation because they presume social interactions will not likely be positive. However, rejection-sensitive individuals expect exclusion in social situations, yet they respond with more hostility to exclusion than less-sensitive individuals (Ayduk et al., 2008; Pfundmair et al., 2015), suggesting accurate expectations may not always offer advantages. Regardless, individuals' expectations of inclusion, and the subsequent effects on relational evaluation, should be considered in future theorizing and research on the effects of social exclusion.

## Author contributions

EW, JW, and MB each contributed to the theoretical arguments in this article.

### Conflict of interest statement

The authors declare that the research was conducted in the absence of any commercial or financial relationships that could be construed as a potential conflict of interest.

## References

[B1] Arriaga X. B. Capezza N. M. Reed J. T. Wesselmann E. D. Williams K. D. (2014). With partners like you, who needs strangers? Ostracism involving a romantic partner. Pers. Relat. 21, 557–569. 10.1111/pere.12048

[B2] Ayduk Ö. Gyurak A. Luerssen A. (2008). Individual differences in the rejection– aggression link in the hot sauce paradigm: the case of rejection sensitivity. J. Exp. Soc. Psychol. 44, 775–782. 10.1016/j.jesp.2007.07.004 20228947 PMC2836513

[B3] Baumeister R. F. Leary M. R. (1995). The need to belong: desire for interpersonal attachments as a fundamental human motivation. Psychol. Bull. 117, 497–529. 10.1037/0033-2909.117.3.497 7777651

[B4] Baumeister R. F. Twenge J. M. Nuss C. K. (2002). Effects of social exclusion on cognitive processes: anticipated aloneness reduces intelligent thought. J. Pers. Soc. Psychol. 83, 817–827. 10.1037/0022-3514.83.4.817 12374437

[B5] Bernstein M. J. Claypool H. M. (2012). Social exclusion and pain sensitivity why exclusion sometimes hurts and sometimes numbs. Pers. Soc. Psychol. Bull. 38, 185–196. 10.1177/0146167211422449 21885860

[B6] Bernstein M. J. Claypool H. M. Young S. G. Tuscherer T. Sacco D. F. Brown C. M. (2013). Never let them see you cry self-presentation as a moderator of the relationship between exclusion and self-esteem. Pers. Soc. Psychol. Bull. 39, 1293–1305. 10.1177/0146167213495281 23861201

[B7] Bernstein M. J. Sacco D. F. Brown C. M. Young S. G. Claypool H. M. (2010a). A preference for genuine smiles following social exclusion. J. Exp. Soc. Psychol. 46, 196–199. 10.1016/j.jesp.2009.08.010

[B8] Bernstein M. J. Sacco D. F. Young S. G. Hugenberg K. Cook E. (2010b). Being “in” with the in-crowd: the effects of social exclusion and inclusion are enhanced by the perceived essentialism of ingroups and outgroups. Pers. Soc. Psychol. Bull. 36, 999–1009. 10.1177/0146167210376059 20693384

[B9] Bernstein M. J. Young S. G. Brown C. M. Sacco D. F. Claypool H. (2008). Adaptive responses to social exclusion: social rejection improves detection of real and fake smiles. Psychol. Sci. 19, 981–983. 10.1111/j.1467-9280.2008.02187.x 19000206

[B10] Böckler A. Hömke P. Sebanz N. (2014). Invisible man: exclusion from shared attention affects gaze behavior and self-reports. Soc. Psychol. Personal. Sci. 5, 140–148. 10.1177/1948550613488951

[B11] Buckley K. E. Winkel R. E. Leary M. R. (2004). Reactions to acceptance and rejection: effects of level and sequence of relational evaluation. J. Exp. Soc. Psychol. 40, 14–28. 10.1016/S0022-1031(03)00064-7

[B12] Bushman B. J. Baumeister R. F. (1998). Threatened egotism, narcissism, self-esteem, and direct and displaced aggression: does self-love or self-hate lead to violence? J. Pers. Soc. Psychol. 75, 219–229. 10.1037/0022-3514.75.1.219 9686460

[B13] Cursan A. Bernstein M. J. Pascual A. Félonneau M. L. (2016). Impact of gendered ingroup/outgroup ostracism on women's academic performances. J. Soc. Psychol. [Epub ahead of print]. 10.1080/00224545.2016.1215966 27454333

[B14] DeWall C. N. Maner J. K. Rouby D. A. (2009). Social exclusion and early-stage interpersonal perception: selective attention to signs of acceptance. J. Pers. Soc. Psychol. 96, 729–741. 10.1037/a0014634 19309198

[B15] Dotan-Eliaz O. Sommer K. L. Rubin Y. S. (2009). Multilingual groups: effects of linguistic ostracism on felt rejection and anger, coworker attraction, perceived team potency, and creative performance. Basic Appl. Soc. Psychol. 31, 363–375. 10.1080/01973530903317177

[B16] Downey G. Feldman S. I. (1996). Implications of rejection sensitivity for intimate relationships. J. Pers. Soc. Psychol. 70, 1327–1343. 10.1037/0022-3514.70.6.1327 8667172

[B17] Gardner W. L. Pickett C. L. Brewer M. B. (2000). Social exclusion and selective memory: how the need to belong influences memory for social events. Pers. Soc. Psychol. Bull. 26, 486–496. 10.1177/0146167200266007

[B18] Geller D. M. Goodstein L. Silver M. Sternberg W. C. (1974). On being ignored: the effects of the violation of implicit rules of social interaction. Sociometry 37, 541–556. 10.2307/2786426

[B19] Gonsalkorale K. Williams K. D. (2007). The KKK won't let me play: ostracism even by a despised outgroup hurts. Eur. J. Soc. Psychol. 37, 1176–1185. 10.1002/ejsp.392

[B20] Goodwin S. A. Williams K. D. Carter-Sowell A. R. (2010). The psychological sting of stigma: the costs of attributing ostracism to racism. J. Exp. Soc. Psychol. 46, 612–618. 10.1016/j.jesp.2010.02.002

[B21] Hales A. H. Williams K. D. Rector J. (in press). Alienating the Audience: How Abbreviations Hamper Scientific Communication. APS Observer.

[B22] Hitlan R. T. Kelly K. M. Schepman S. Schneider K. T. Zárate M. A. (2006). Language exclusion and the consequences of perceived ostracism in the workplace. Group Dyn. Theor. Res. Pract. 10:56 10.1037/1089-2699.10.1.56

[B23] Jones E. E. Carter-Sowell A. R. Kelly J. R. (2011). Participation matters: psychological and behavioral consequences of information exclusion in groups. Group Dyn. Theor. Res. Pract. 15, 311–325. 10.1037/a0025547

[B24] Jones E. E. Carter-Sowell A. R. Kelly J. R. Williams K. D. (2009). I'm out of the loop': Ostracism through information exclusion. Group Process. Intergroup Relat. 12, 157–174. 10.1177/1368430208101054

[B25] Kerr N. L. Levine J. M. (2008). The detection of social exclusion: evolution and beyond. Group Dyn. Theor. Res. Pract. 12, 39–52. 10.1037/1089-2699.12.1.39

[B26] Klages S. V. Wirth J. H. (2014). Excluded by laughter: laughing until it hurts someone else. J. Soc. Psychol. 154, 8–13. 10.1080/00224545.2013.843502 24689333

[B27] Koudenburg N. (2014). Conversational Flow. Doctoral dissertation, University of Groningen.

[B28] Koudenburg N. Postmes T. Gordijn E. H. (2011). Disrupting the flow: how brief silences in group conversations affect social needs. J. Exp. Soc. Psychol. 47, 512–515. 10.1016/j.jesp.2010.12.006

[B29] Koudenburg N. Postmes T. Gordijn E. H. (2013). Conversational flow promotes solidarity. PLoS ONE 8:e78363. 10.1371/journal.pone.0078363 24265683 PMC3827030

[B30] Leary M. R. (1990). Responses to social exclusion: social anxiety, jealousy, loneliness, depression, and low self-esteem. J. Soc. Clin. Psychol. 9, 221–229. 10.1521/jscp.1990.9.2.221

[B31] Leary M. R. (1999). Making sense of self-esteem. Curr. Dir. Psychol. Sci. 8, 32–35. 10.1111/1467-8721.00008

[B32] Leary M. R. Baumeister R. F. (2000). The nature and function of self-esteem: sociometer theory, in Advances in Experimental Social Psychology, Vol. 32, ed Zanna M. P. (San Diego, CA: Academic Press), 1–62.

[B33] Leary M. R. Cottrell C. A. Phillips M. (2001). Deconfounding the effects of dominance and social acceptance on self-esteem. J. Pers. Soc. Psychol. 81, 898–909. 10.1037/0022-3514.81.5.898 11708565

[B34] Lieberman M. D. (2013). Social: Why Our Brains are Wired to Connect. New York, NY: Crown Publishers.

[B35] Maner J. K. DeWall C. N. Baumeister R. F. Schaller M. (2007). Does social exclusion motivate interpersonal reconnection? Resolving the “porcupine problem.” J. Pers. Soc. Psychol. 92, 42–55. 10.1037/0022-3514.92.1.42 17201541

[B36] Mead N. L. Baumeister R. F. Stillman T. F. Rawn C. D. Vohs K. D. (2011). Social exclusion causes people to spend and consume strategically in the service of affiliation. J. Cons. Res. 37, 902–919. 10.1086/656667

[B37] Nezlek J. B. Wesselmann E. D. Wheeler L. Williams K. D. (2012). Ostracism in everyday life. Group Dyn. Theor. Res. Pract. 16, 91–104. 10.1037/a0028029

[B38] Pfundmair M. DeWall C. N. Fries V. Geiger B. Krämer T. Krug S. (2015). Sugar or spice: using I^3^ metatheory to understand how and why glucose reduces rejection-related aggression. Aggress. Behav. 41, 537–543. 10.1002/ab.21593 26198908

[B39] Pickett C. L. Gardner W. L. (2005). The social monitoring system: enhanced sensitivity to social cues as an adaptive response to social exclusion, in The Social Outcast: Ostracism, Social Exclusion, Rejection, and Bullying, eds Williams K. D. Forgas J. P. von Hippel W. (New York, NY: Psychology Press), 213–226.

[B40] Pickett C. L. Gardner W. L. Knowles M. (2004). Getting a cue: the need to belong and enhanced sensitivity to social cues. Pers. Soc. Psychol. Bull. 30, 1095–1107. 10.1177/0146167203262085 15359014

[B41] Riva P. Eck J. (2016). The many faces of social exclusion, in Social Exclusion: Psychological Approaches to Understanding and Reducing Its Impact, eds Riva P. Eck J. (Springer International Publishing), 9–15.

[B42] Riva P. Montali L. Wirth J. H. Curioni S. Williams K. D. (2016). Chronic social exclusion and evidence for the resignation stage: an empirical investigation. J. Soc. Pers. Relat. [Epub ahead of print]. 10.1177/0265407516644348

[B43] Riva P. Wesselmann E. D. Wirth J. H. Carter-Sowell A. R. Williams K. D. (2014). When pain does not heal: the common antecedents and consequences of chronic social and physical pain. Basic Appl. Soc. Psych. 36, 329–346. 10.1080/01973533.2014.917975

[B44] Rudert S. C. Greifeneder R. (2016). When it's okay that I don't play: social norms and the situated construal of social exclusion. Pers. Soc. Psychol. Bull. 42, 955–969. 10.1177/0146167216649606 27229676

[B45] Sacco D. F. Wirth J. H. Hugenberg K. Chen Z. Williams K. D. (2011). The world in black and white: ostracism enhances the categorical perception of social information. J. Exp. Soc. Psychol. 47, 836–842. 10.1016/j.jesp.2011.03.001

[B46] Shilling A. A. Brown C. M. (2016). Goal-driven resource redistribution: an adaptive response to social exclusion. Evol. Behav. Sci. 10, 149–167. 10.1037/ebs0000062

[B47] Smart Richman L. Leary M. R. (2009). Reactions to discrimination, stigmatization, ostracism, and other forms of interpersonal rejection: a multimotive model. Psychol. Rev. 116, 365–383. 10.1037/a0015250 19348546 PMC2763620

[B48] Somerville L. H. Heatherton T. F. Kelley W. M. (2006). Anterior cingulate cortex responds differentially to expectancy violation and social rejection. Nat. Neurosci. 9, 1007–1008. 10.1038/nn1728 16819523

[B49] Spoor J. Williams K. D. (2007). The evolution of an ostracism detection system, in The Evolution of the Social Mind: Evolutionary Psychology and Social Cognition, eds Forgas J. P. Haselton M. von Hippel W. (New York, NY: Psychology Press), 279–292.

[B50] Tuscherer T. Sacco D. F. Wirth J. H. Claypool H. M. Hugenberg K. Wesselmann E. D. (2016). Responses to exclusion are moderated by its perceived fairness. Eur. J. Soc. Psychol. 46, 280–293. 10.1002/ejsp.2152

[B51] Twenge J. M. Baumeister R. F. Tice D. M. Stucke T. S. (2001). If you can't join them, beat them: effects of social exclusion on aggressive behavior. J. Pers. Soc. Psychol. 81, 1058–1069. 10.1037/0022-3514.81.6.1058 11761307

[B52] Twenge J. M. Campbell W. K. (2003). “Isn't it fun to get the respect that we're going to deserve?” Narcissism, social rejection, and aggression. Pers. Soc. Psychol. Bull. 29, 261–272. 10.1177/0146167202239051 15272953

[B53] Wesselmann E. D. Butler F. A. Williams K. D. Pickett C. L. (2010). Adding injury to insult: unexpected rejection leads to more aggressive responses. Aggress. Behav. 36, 232–237. 10.1002/ab.20347 20540160

[B54] Wesselmann E. D. Cardoso F. D. Slater S. Williams K. D. (2012a). To be looked at as though air: civil attention matters. Psychol. Sci. 23, 166–168. 10.1177/0956797611427921 22246319

[B55] Wesselmann E. D. Grzybowski M. R. Steakley-Freeman D. M. DeSouza E. R. Nezlek J. B. Williams K. D. (2016). Social exclusion in everyday life, in Social Exclusion: Psychological Approaches to Understanding and Reducing Its Impact, eds Riva P. Eck J. (Springer International Publishing), 3–23.

[B56] Wesselmann E. D. Nairne J. S. Williams K. D. (2012b). An evolutionary social psychological approach to studying the effects of ostracism. J. Soc. Evol. Cult. Psychol. 6, 309–328. 10.1037/h0099249

[B57] Wesselmann E. D. Ren D. Williams K. D. (2015). Motivations for responses to ostracism. Front. Psychol. 6:40 10.3389/fpsyg.2015.00040 25691876 PMC4315012

[B58] Wesselmann E. D. Wirth J. H. Pryor J. B. Reeder G. D. Williams K. D. (2013). When do we ostracize? Soc. Psychol. Personal. Sci. 4, 108–115. 10.1177/1948550612443386

[B59] Williams K. D. (2009). Ostracism: effects of being excluded and ignored, in Advances in Experimental Social Psychology, ed Zanna M. (New York, NY: Academic Press), 275–314.

[B60] Williams K. D. Cheung C. K. Choi W. (2000). Cyberostracism: effects of being ignored over the Internet. J. Pers. Soc. Psychol. 79, 748–762. 10.1037/0022-3514.79.5.748 11079239

[B61] Williams K. D. Wesselmann E. D. (2011). The link between ostracism and aggression, in The Psychology of Social Conflict and Aggression, eds Forgas J. P. Kruglanski A. W. Williams K. D. (New York, NY: Psychology Press), 37–51.

[B62] Wirth J. H. Bernstein M. J. Wesselmann E. D. LeRoy A. S. (2017). Social cues establish expectation of rejection and affect the response to being rejected. Group Process. Intergroup Relat. 20, 32–51. 10.1177/1368430215596073

[B63] Wirth J. H. Sacco D. F. Hugenberg K. Williams K. D. (2010). Eye gaze as relational evaluation: averted eye gaze leads to feelings of ostracism and relational devaluation. Pers. Soc. Psychol. Bull. 36, 869–882. 10.1177/0146167210370032 20505162

[B64] Wirth J. H. Williams K. D. (2009). “They don't like our kind”: consequences of being ostracized while possessing a group membership. Group Process. Intergroup Relat. 12, 111–127. 10.1177/1368430208098780

